# Optic disk melanocytoma associated with polypoidal choroidal vasculopathy lesions, after combination treatment of photodynamic therapy and intavitreal aflibercept (Eylea), a case report

**DOI:** 10.1186/s12886-018-0927-7

**Published:** 2018-10-12

**Authors:** Alexandros Rouvas, Nikolaos S Gouliopoulos, Marilita M Moschos, Panagiotis Theodossiadis

**Affiliations:** 1 2nd Department of Ophthalmology, University of Athens Medical School, ‘Attikon’ General Hospital Athens, 1 Rimini Str, Haidari, 12462 Athens, Greece; 2 1st Department of Ophthalmology, University of Athens Medical School, ‘G. Genimmatas’ General Hospital Athens, 154 Mesogion Avenue, Holargos, Athens, Greece

**Keywords:** Polypoidal choroidal vasculopathy, Optic disc melanocytoma, Aflibercept, Photodynamic therapy

## Abstract

**Background:**

We report a rare case of a woman with optic disk melanocytoma (ODMC) in conjunction with polypoidal choroidal vasculopathy (PCV). We also present, for the first time in literature, the clinical and morphological outcomes of the applied treatment, consisting of a session of photodynamic therapy (PDT) and three monthly intravitreal aflibercept injections.

**Case presentation:**

An 83-year-old Greek woman, complaining for visual decline at her left eye, referred to our department and was diagnosed with ODMC associated with PCV. At presentation, best corrected visual acuity (BCVA) was 2/10, fundus examination revealed a pigmented lesion covering partially the optic nerve head and extending into the peripapillary choroid and retina, while hard exudates were observed temporal to it. Blocked hypofluorescence in the area covered by the lesion and diffuse hyperfluorescence at its temporal rim were shown by fluorescein angiography (FA). Indocyanine green angiography (ICGA) identified 3 hyperfluorescent polypoidal lesions arising from the choroidal vasculature. Optical coherence tomography (OCT) revealed subretinal fluid and retinal pigment epithelium detachment (RPE) at the region corresponding to polyps. The treatment included a PDT session combined with 3 monthly intravitreal aflibercept injections. Three months since the treatment initiation, new BCVA was 5/10, ICGA demonstrated total polyps occlusion, while OCT detected RPE detachment without subretinal fluid. Ten months later, ODMC was stable, BCVA rose to 7/10, no polyps were present, and total resolution of RPE detachment was achieved.

**Conclusions:**

This is the first case report of PCV coexisting with ODMC, presenting both ICGA and OCT findings, and the applied treatment and its outcomes. Furthermore, we demonstrated that PDT combined with intravitreal aflibercept injections seems to be a promising treatment for PCV.

## Background

Optic disc melanocytoma (ODMC) is a rare ophthalmic tumor arising from melanocytes, and is considered to be a variant of the melanocytic nevus [1]. ODMC is a benign and deeply pigmented lesion, which obscures partially or completely the optic disc, extending frequently into the adjacent retina and/or choroid [1, 2]. Its pathogenesis is unknown, being either a congenital or an acquired clinical entity. Histologically, melanocytoma comprises round or oval and intensely pigmented nevus cells with benign features (abundant cytoplasm, small nuclei, and inconspicuous nucleoli) [2]. ODMC is usually unilateral and diagnosed in middle-aged adults. It is slightly more common in women, while almost 67% of cases are regarding white people [3]. ODMC is usually asymptomatic, however approximately 25% of the patients suffer by mild or severe visual deterioration, due to mild retinal exudation (involving the fovea) or neuroretinitis from tumor necrosis [1, 3, 4].

Polypoidal choroidal vasculopathy (PCV), first described by Yannuzzi et al. [5], is a clinical entity of unknown etiology, which is characterized by polypoidal aneurysmal dilatations of the inner choroidal vascular network [6]. PCV is clinically visible as subretinal reddish orange spherical lesions, which tend to leak extensively, producing subretinal hemorrhages and exudates, and retinal pigment epithelium (RPE) detachments [5]. Indocyanine green angiography (ICGA) is the gold standard examination for its detection and definitive diagnosis [7]. Although PCV may be asymptomatic, the polyps frequently leak and the macula is affected by serosanguinous complications, causing visual impairment [8]. Without proper and timely treatment, 50% of patients would suffer from macular degeneration and irreversible visual loss due to recurrent episodes of bleeding and leaking of the polypoid vessels [9].

In this report we describe a case of a woman with ODMC along with PCV. To the best of our knowledge it has been previously described only by Bartlett et al. and El-Haddad et al. [10, 11]. However, our report is the first in literature in which the applied treatment and the clinical and morphological outcomes of the provided treatment in 3 and 13 months follow up are presented.

## Case presentation

An 83-year-old Greek woman, without any prior significant medical history, referred to the outpatient department of the 2nd Department of Ophthalmology of the Medical School of Athens, complaining for visual deterioration at her left eye (OS). At presentation she underwent a complete ophthalmological examination. Best corrected visual acuity (BCVA) was 10/10 in her right eye (OD) and 2/10 in OS. Slit lamp examination did not identify any abnormalities in the anterior segments in both eyes. Her intraocular pressure was normal bilaterally.

**Fig. 1:**
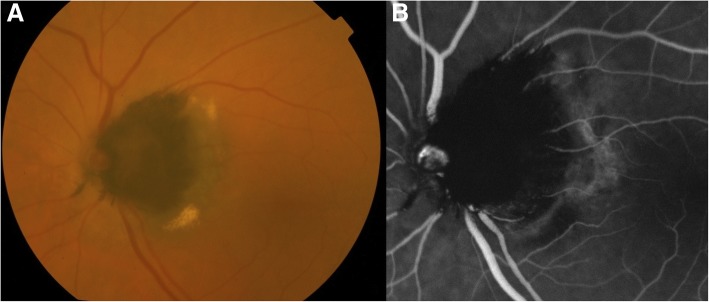
Fundus photography (**a**) and fluorescein angiography (**b**) of the left eye at baseline examination. **a** A deeply pigmented lesion, covering partially the optic nerve head and extending temporal into the peripapillary retina and choroid is consistent with optic disk melanocytoma (ODMC). Note the hard exudates temporal to ODMC and the pigmented spicules along the vessels. **b** Fluorescein angiography demonstrated diffuse blocked hypofluerescence in the area covered by ODMC, with diffuse hyperfluorescence at its temporal rim

Funduscopy and fundus photography revealed an unremarkable retina in OD. However, in OS a dark brown lesion, with “fuzzy” borders was identified, which covered partially the optic nerve head and extended temporal into the adjacent choroid and retina (Fig. 1a). Furthermore, hard exudates were observed temporal to the aforementioned pigmented lesion.

**Fig. 2:**
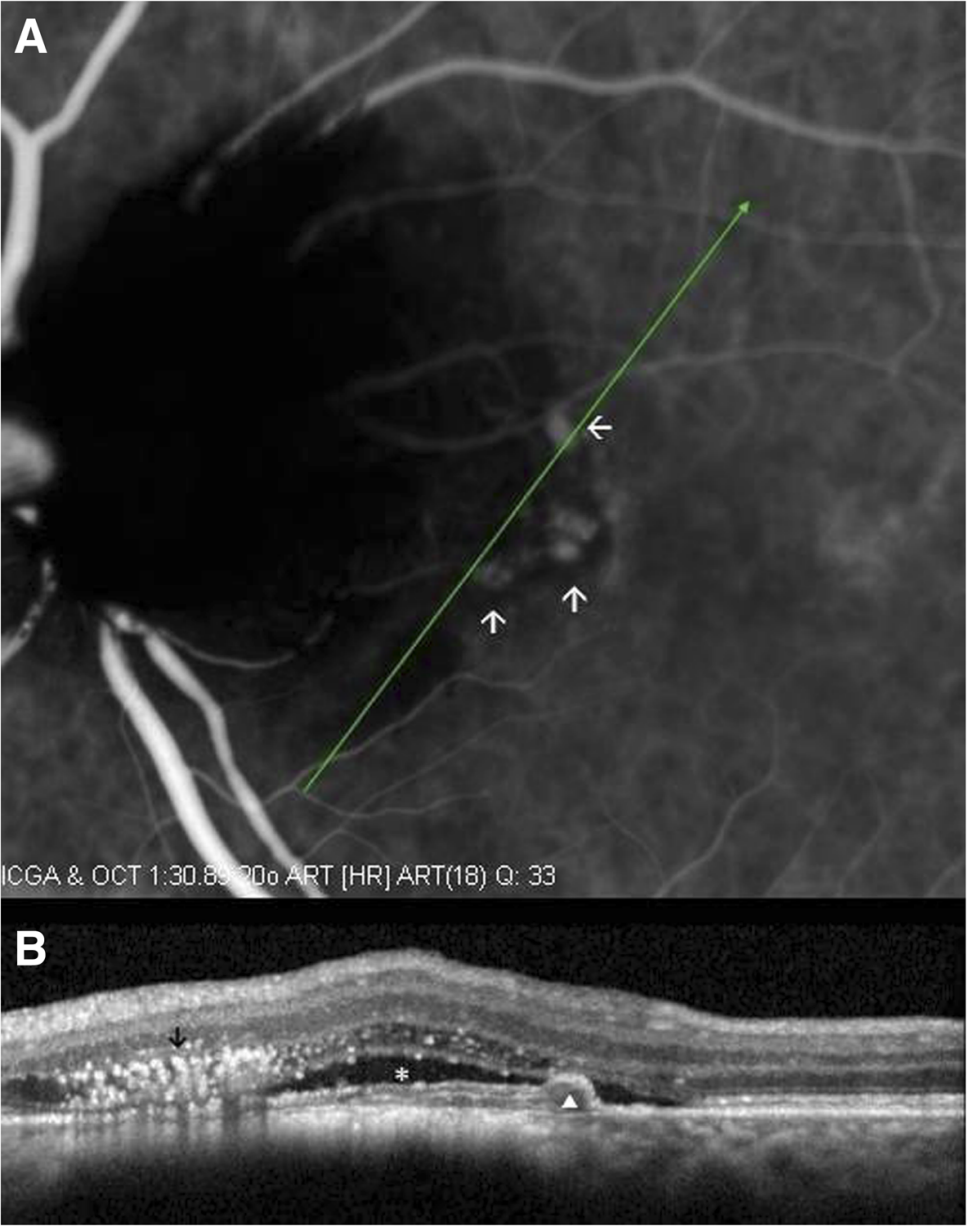
ICGA (**a**) and OCT (**b**) examination of the left eye at baseline. **a** Three hyperfluorescent polypoidal lesions (white arrows) arising from the choroidal circulation were identified. **b** In OCT, the ODMC was shown as a gradually sloped nodular elevation, with hyperreflective anterior surface and dense posterior shadowing (black arrow). Subretinal fluid (white *), RPE detachment (white arrow-head), and a round protrusion beneath the posterior surface of the detached RPE (white arrow-head) were also detected at the region corresponding to polyps in ICGA

Fluorescein angiography (FA) (Fig. 1b) and ICGA (Fig. 2a) showed diffuse blocked hypofluerescence in all phases in the area covered by the pigmented lesion, with diffuse hyperfluorescence at the temporal rim of the lesion. As well, ICGA detected 3 hyperfluorescent polypoidal lesions arising from the choroidal circulation (Fig. 2a).

**Fig. 3:**
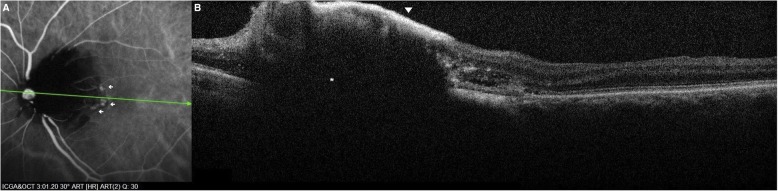
Baseline ICGA examination (**a**) and OCT scan (**b**) passing through the center of the tumor and one of the accompanied polyps. **a** Diffuse blocked hypofluorescence is detected in the area covered by ODMC. Polyps correspond to the hyperfluorescent polypoidal lesions (white arrows) arising from the choroidal circulation. **b** ODMD is shown as a gradually sloped elevation, with hyperreflective anterior surface (white arrow head), and abrupt, dense posterior shadowing portraying an optical mass (*)

The pigmented lesion was shown in optical coherence tomography (OCT) (SPECTRALIS, Heidelberg Engineering, Heidelberg, Germany) as a gradually sloped nodular elevation, with hyperreflective anterior surface and dense posterior shadowing. OCT also revealed subretinal fluid, RPE detachment, and a round protrusion attached beneath the posterior surface of the detached RPE, at the site corresponding to polypoidal lesions in ICGA (Figs. 2b and 3).

A diagnosis of PCV associated with ODMC was made.

**Fig. 4:**
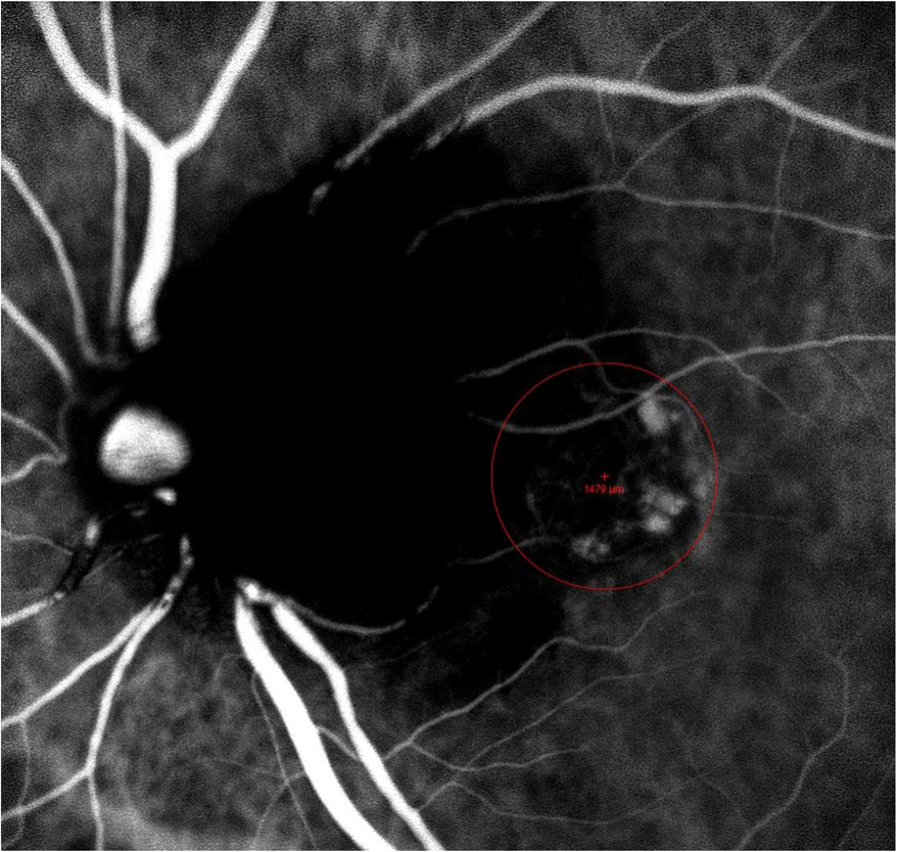
Based on ICGA findings, the spot diameter of PDT treatment was ~ 1500 μm

The treatment strategy included a session of verteporfin photodynamic therapy (PDT) in combination with 3 monthly intravitreal aflibercept injections. PDT was performed according to the standard TAP guidelines [12]. Fifteen minutes after the infusion of verteporfin (dose of 6 mg/m^2^ body surface area), its irradiation was performed using an ocular photoactivation diode and a laser-linked slit lamp. The treatment spot diameter, based on the ICGA findings, was approximately 1500 μm (Fig. 4) and it was targeted against the polyps and not the surrounding branching vascular network. The intravitreal injections of 2.0 mg aflibercept (Eylea, Bayer Healthcare, Germany), were performed under standard sterile conditions, while topical antibiotics were applied 4 times per day for 2 days after the injection.

PDT session was performed 7 days after the first intravitreal aflibercept injection. One and two months later, the several injections were applied.

**Fig. 5:**
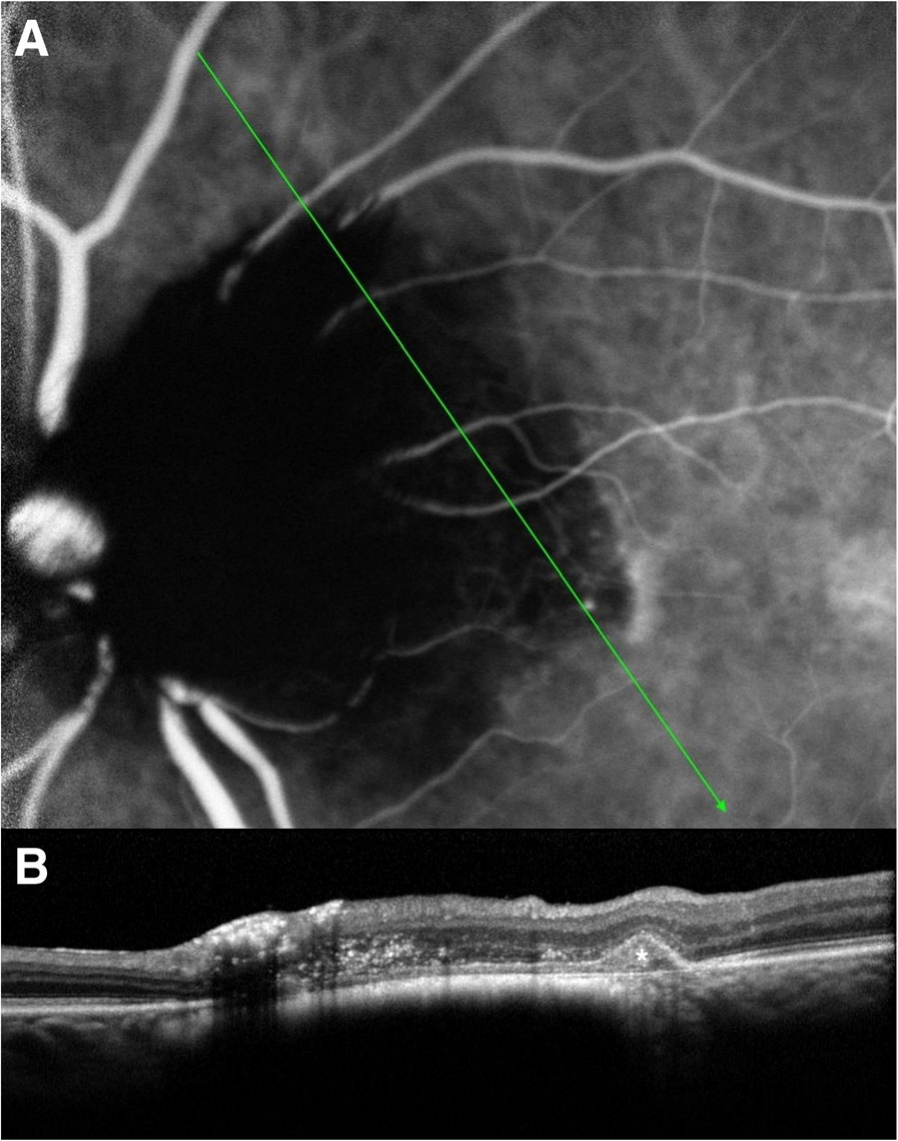
ICGA (**a**) and OCT (**b**) findings, 3 months after the treatment initiation. **a** No polyps were detected by ICGA. **b** In OCT RPE detachment was present (*), whereas subretinal fluid was not evident

Three months after the treatment initiation, significant improvement was observed. Patient’s new BCVA was 5/10, while ICGA demonstrated total polyp regression (Fig. 5a). In OCT RPE detachment was present, whereas subretinal fluid was not evident (Fig. 5b).

**Fig. 6:**
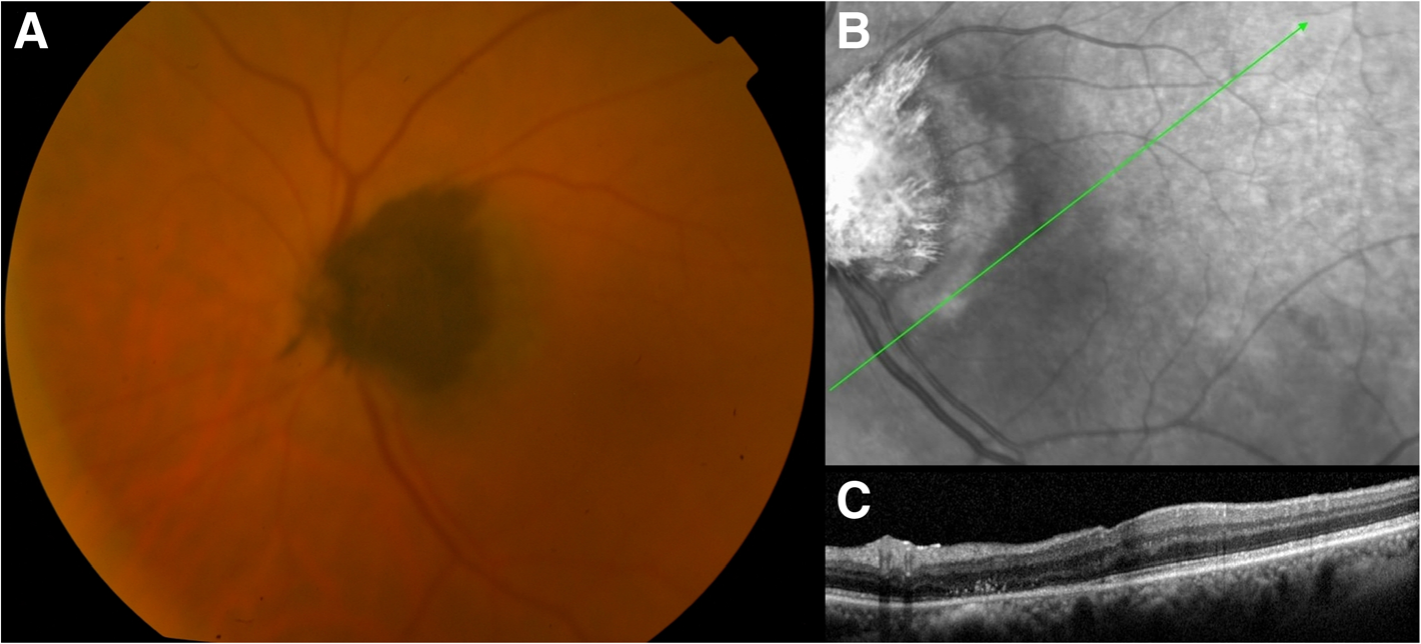
Fundus photography (**a**), infrared fundus photography (**b**) and OCT examination (**c**), 13 months after the treatment initiation. ODMC remained stable, no polyps were detected, and total resolution of RPE detachment was achieved

Thirteen months since the beginning of the treatment, the patient was re-examined. ODMC remained stable, her BCVA rose to 7/10, no polyps were detected, while total resolution of RPE detachment was achieved (Fig. 6).

## Discussion

This is the first report in literature presenting a rare clinical case of ODMC coexisting with PCV, in which the applied treatment and its clinical outcomes are described, and the findings of SPECTRALIS OCT, which depicts the clinical lesions with more accuracy, are provided.

Several therapeutic strategies have been applied in PCV treatment. PDT has been proven to be effective in inducing polyps occlusion and reducing exudative phenomena in PCV eyes, improving thus visual acuity [13–15]. However, an elevated risk of subretinal or sub-RPE hemorrhage exists, because PDT is usually guided both on the polyps and the surrounding network [14–16]. Based on previous reports, according to which PDT has no or little effect on the branching vascular network [17], we targeted the diode laser spot only on the polyps, avoiding thus the aforementioned adverse effects. Rouvas et al. [8] demonstrated that PDT alone was superior to intravitreal ranibizumab (IVR) injections combined or not with PDT, in achieving better functional and anatomical features over a 12 months period. The EVEREST II study suggested that PDT in combination with IVR injections, is superior compared to IVR monotherapy in achieving complete polyps regression and significant visual gains in PCV patients over a 12 months period [13].

Aflibercept is a recombinant fusion protein, binding to all isomers of vascular endothelial growth factor (VEGF)-A family, to VEGF-B and to placental growth factor [18, 19]. Its efficacy in resolving the polyps seems to be theoretically greater than ranibizumab, because the last one binds only to VEGF-A and has decreased binding affinity for VEGF compared to aflibercept [18, 19]. Furthermore, it has been suggested that aflibercept is more effective in visual improvement compared to ranibizumab for sub-RPE lesions [20]. The PLANET study [21] and Yamamoto et al. [22] showed that intravitreal aflibercept injections as monotherapy resulted in excellent visual and structural benefits, while Morimoto et al. [23] demonstrated that a treat-and-extend regimen with intravitreal aflibercept injections may be efficient for improvement of visual acuity and exudative phenomena in PCV eyes.

Our treatment strategy, consisting of PDT along with three intravitreal aflibercept injections, seems to be effective, since our patient experienced significant vision and anatomical gains. Polyps closed completely, their recurrence was prevented in a thirteen-month period, while resolution of RPE detachment occurred.

As for ODMC, no changes were detected during the 12-month follow up. ODMC is regarded to be a benign and stable lesion, while only 1–2% of the cases undergo a malignant transformation [3]. In the literature, no adverse effects of PDT on ODMC have been reported [24].

## Conclusions

To the best of our knowledge, no previously published data describe a case of PCV coexisting with ODMC, displaying both ICGA and OCT findings. Furthermore, this is also the first report in literature that any form of treatment is applied for PCV associated with ODMC. Last but not least, we present the combination of PDT and intravitreal aflibercept injections as a promising treatment for PCV.

## Abbreviations

BCVA: Best corrected visual acuity
FA: Fluorescein angiography
ICGA: Indocyanine green angiography
IVR: Intravitreal ranibizumab
OCT: Optical coherence tomography
OD: Right eye
ODMC: Optic disc melanocytoma
OS: Left eye
PCV: Polypoidal choroidal vasculopathy
PDT: Photodynamic therapy
RPE: Retinal pigment epithelium
VEGF: Vascular endothelial growth factor
